# Associations of Optic Nerve Head and Parapapillary Atrophy Changes With Retinal Nerve Fiber Layer and Ganglion Cell Complex Thinning in Healthy Eyes

**DOI:** 10.1167/tvst.15.10.34

**Published:** 2026-10-23

**Authors:** Caixia Li, Yanyan Cheng, Liqin Song, Mengyang Li, Xiaohua Pan, Fei Xiang, Yi Wang, Junying Ren, Zehua Wang, Yawen Li, Li Guo, Hongyu Wei, Xian Shao, Shuning Li

**Affiliations:** 1 Shaanxi Eye Hospital, Xi'an People's Hospital (Xi'an Fourth Hospital), Xi'an, Shaanxi, China; 2 Hebei Eye Hospital, Hebei Provincial Key Laboratory of Ophthalmology, Hebei Provincial Clinical Research Center for Eye Diseases, Xingtai, Hebei, China; 3 Chenzhou Aier Eye Hospital, Chenzhou, Hunan, China; 4 Beijing Tongren Eye Center, Beijing Tongren Hospital, Beijing Ophthalmology & Visual Sciences Key Laboratory, Capital Medical University, Beijing, China; 5 Shenzhen Eye Hospital, Shenzhen Eye Institute, Jinan University, Shenzhen, Guangdong, China; 6 Shenzhen Eye Hospital, Shenzhen Eye Center, Southern Medical University, Shenzhen, Guangdong, China; 7 Shijiazhuang People's Hospital, Shijiazhuang, Hebei, China; 8 Handan City Eye Hospital (The Third Hospital of Handan), Handan, Hebei, China; 9 The First Affiliated Hospital of Kunming Medical University, Kunming, Yunnan, China

**Keywords:** retinal nerve fiber layer, ganglion cell complex, optic nerve head, glaucoma, follow-up

## Abstract

**Purpose:**

To determine whether retinal nerve fiber layer (RNFL) and ganglion cell complex (GCC) thinning occurs in association with changes in the optic nerve head (ONH) and parapapillary atrophy (PPA) over five years’ follow-up.

**Methods:**

This longitudinal observational study recruited participants aged ≥50 years. Participants were classified into ONH/PPA changed and ONH/PPA unchanged groups based on changes in ONH shape, the position of retinal vessels, and PPA morphology. Changes in RNFL and GCC were compared between the two subgroups. Linear regression models were used to evaluate the associations between changes in RNFL and GCC thickness and both ocular and systemic factors.

**Results:**

A total of 149 healthy eyes were included. During the five-year follow-up period, both RNFL and GCC thickness exhibited significant thinning. Specifically, the RNFL thinned by 1.59 µm, and the GCC thinned by 1.11 µm. Significant differences were observed in the change of the inferior RNFL between the two subgroups (*P*_adj_ = 0.044), whereas no statistically significant differences were found for other parameters. The change in inferior RNFL was independently associated with baseline axial length (AL) (*P* = 0.025), change in AL (*P* = 0.003), change in disc area (*P* < 0.001) and change in ONH/PPA (*P* = 0.045); whereas the change in average GCC was not significantly associated with any of the evaluated parameters.

**Conclusions:**

Significant longitudinal changes in RNFL and GCC thickness were observed in healthy eyes. GCC parameters appear to be unrelated to morphological changes of the ONH and PPA, which may facilitate the early detection of glaucoma and its progression in individuals with age-related ONH/PPA alterations.

**Translational Relevance:**

This study provides clinicians with a key insight: GCC parameters may offer greater clinical utility than RNFL parameters for detecting early glaucoma and monitoring disease progression in individuals with age-related structural alterations of the ONH and PPA region.

## Introduction

Glaucoma is a leading cause of preventable irreversible blindness globally.^1^ Monitoring disease progression is a cornerstone of effective glaucoma management.^2^ Current evidence supports a broadly linear association between structural and functional decline in glaucoma; however, owing to inherently higher test-retest variability in standard automated perimetry compared with optical coherence tomography, structural abnormalities—particularly retinal nerve fiber layer (RNFL) thinning—are often detectable before corresponding functional deficits reach statistical significance.^3^^,^^4^ Accurate identification of structural changes allows clinicians to implement timely diagnosis and intervention in early glaucoma, thereby reducing the risk of visual impairment in affected individuals. Optical coherence tomography (OCT) application in the eye is a noninvasive cross-sectional imaging modality with good measurement repeatability and has become an important diagnostic tool in glaucoma assessment.^5^^,^^6^ Existing studies have demonstrated the importance of RNFL and ganglion cell complex (GCC) parameters measured by OCT in the early screening of glaucoma.^7^^–^^10^ However, because of aging, RNFL and GCC are thinning physiologically; furthermore, with aging, the optic nerve head (ONH) in some people will experience shape changing or enlargement of parapapillary atrophy (PPA). It is still not certain whether RNFL and GCC measurement are affected by the changing shape of ONH and PPA during follow-up, which is meaningful for using OCT parameters to assess glaucoma progress during follow-up. The aim of our study is to investigate the effects of changes in ONH structure and PPA on changes in RNFL and GCC parameters in healthy eyes of individuals aged 50 years and older over a five-year follow-up.

## Methods

This is a longitudinal observational study conducted in the Daxing District of Beijing, China, in October 2018 and July 2023. The study has received approval from the Clinical Research Ethics Committee of Beijing Tongren Hospital, Capital Medical University. All study participants provided written informed consent. This study adhered to the principles of the Helsinki Declaration.

### Study Participants

This study has been registered with the Chinese Clinical Trial Registry (registration number: ChiCTR2200062375). In 2018 and 2023, a total of 1419 and 1356 individuals participated in the study, respectively. Among them, 380 participants aged ≥50 years underwent follow-up. These subjects were enrolled in an observational, longitudinal study aimed at investigating changes in RNFL and GCC thickness in healthy eyes. The inclusion criteria were as follows: (1) age ≥ 50 years, (2) intraocular pressure ≤ 21 mm Hg, (3) spherical equivalent refractive error between −6 and + 6 diopters, and (4) normal appearance of the ONH, including a vertical cup-to-disc ratio (VCDR) < 0.7 and a VCDR difference between both eyes < 0.2. The exclusion criteria were: (1) family history of glaucoma, (2) history of laser, refractive, or intraocular surgery, (3) presence of glaucoma or suspected glaucoma, optic nerve or retinal pathology, epiretinal membrane, history of intraocular inflammation, trauma, tumor, or optic nerve abnormalities, and (4) overall imaging quality index < 6 or severe artifacts in OCT images. Finally, a total of 149 participants meeting the above inclusion and exclusion criteria were included in the study. Because of time constraints, OCT examinations in 2023 were performed only on the better-seeing eye of each participant, and this eye was consistently matched with its corresponding 2018 examination data. Only one eye per participant was selected for image processing and data analysis.

### Clinical Examinations

All participants underwent two comprehensive systemic and ophthalmic examinations, including assessments of age, sex, height, weight, systolic blood pressure (SBP), diastolic blood pressure (DBP), pulse, blood glucose, visual acuity (VA), spherical equivalent refractive error (SER) (Topcon KR-800; Topcon Corporation, Tokyo, Japan), intraocular pressure (IOP) (measured with Topcon CT-80 in 2018 [Topcon Medical Systems, Inc., Oakland, NJ, USA] and NIDEK in 2023 [Nidek, Tokyo, Japan]), axial length (AL) (measured with Lenstar LS900; Haag Streit, Bern, Switzerland), central corneal thickness (CCT) (measured with Lenstar LS900; Haag Streit), as well as measurements of the disc area (DA), cup area (CA), rim area (RA), vertical disc diameter (VDR), VCDR, and maximum cup depth (MCD) obtained using a fundus camera (Kowa Nonmyd WX, Tokyo, Japan). Additionally, RNFL and GCC thickness measurements were obtained using an 80 kHz RTVue XR OCT scanner (with AngioVue software version 2017.1; Optovue, Inc., Fremont, CA, USA). Experienced glaucoma specialists (SNL and MYL) performed slit-lamp examinations (Haag Streit) without dilation, bilateral optic disc assessments, and gonioscopy (Ocular Technology, Inc., Goleta, CA, USA). Questionnaires were administered by dedicated certified technicians and a clinical team of doctors. The IOP and ocular biometry parameters were measured three times, and the average value was taken for statistical analysis.

### OCT Imaging

The RNFL and GCC thickness were measured using a non-mydriatic spectral-domain OCT device (SD-OCT) (Optovue; Optovue, Inc.) equipped with motion-tracking technology for image acquisition, as detailed in our prior study.^11^ Scanning was performed by a trained operator (CXL). Subsequently, an automatic placement of a measurement circle with a diameter of 3.4 mm was placed around the optic disc to calculate the RNFL thickness. The GCC thickness measurement is performed at a fixed scanning range of 7 mm × 7 mm, with the center located 1 mm temporal to the fovea. Follow-up scans were automatically aligned with baseline scans based on the retinal vasculature trajectory to ensure measurements from the same location. Images with a signal strength <6, missing data, poor centration, or segmentation errors were excluded from this study.

OCT-derived retinal images are subject to dual magnification effects arising from both the imaging system and the eye's intrinsic optical properties. To correct for this magnification artifact, we integrated baseline and follow-up AL measurements with the Littmann and the modified Bennett formula to calculate the actual size of ocular parameters. The Littmann formula is expressed as *D_t_ = p × q × D_m_*, where *D_t_* represents the true distance, *D_m_* denotes the measured distance, *p* represents the magnification factor attributable to the imaging system, and *q* denotes the magnification due to the eye.^12^ The factor *q* is calculated via the Bennett formula: *q = 0.01306 × (AL−1.82)*, where AL denotes axial length and the constant 1.82 corresponds to the distance from the corneal apex to the second principal plane. For our imaging system, the true distance is equivalent to the measured distance (*D_t_ = D_m_*) at an AL of 23.82 mm,^13^ enabling direct calculation of factor *P* via the Bennett formula. When *D_t_ = D_m_*, then *p = 1/q* and, therefore, *p = 1/ [0.01306(23.82-1.82)] = 3.48*. Thus the magnification factor of the image should be corrected by: *D_t_= 3.48 × [0.01306 × (AL−1.82)] × D_m_.* We applied both the Littmann correction method and the modified Bennett formula to correct the AL measurements obtained at baseline and follow-up.

### Optic Disc Stereo Imaging

Stereoscopic fundus images of optic disc were acquired using a non-mydriatic stereoscopic fundus camera (Kowa Nonmyd WX), as described in detail in our previous study.^11^ Kim et al.^14^ compared baseline and follow-up optic disc images by using templates generated from baseline photographs of each patient, with the vascular contour around the ONH as a reference. Based on the shape of the optic disc, the position of the optic disc edge relative to the adjacent retinal vessels, any changes in the surrounding area of the ONH were determined. This study employed Fiji (ImageJ 1.54f; National Institutes of Health, Bethesda, MD, USA) to manually delineate anatomical landmarks on baseline fundus photographs for each participant, including the optic disc margin, peripapillary vascular courses, and PPA morphology. Using the manually delineated edges of the optic disc, adjacent retinal vessel courses, and PPA contours as references, we compared their overlap with follow-up fundus images (Fig. 1) and qualitatively classified participants into two groups: the ONH/PPA changed group (72 eyes) and the ONH/PPA unchanged group (77 eyes). We conducted two independent rounds of fundus image labeling and grouping, both performed by the same trained examiner (CXL). We further used Cohen's kappa coefficient to assess the consistency between two independent labeling and grouping results, which showed a kappa value of 0.946 (*P* < 0.001), indicating excellent agreement.

### Statistical Analysis

All statistical analyses were conducted using IBM SPSS Statistics 25.0 software (SPSS, Inc., Chicago, IL, USA). The Kolmogorov–Smirnov test was used to assess the normality of continuous variables. Quantitative data were presented as mean and standard deviation (or median and interquartile range), whereas qualitative data were expressed as percentages. Cohen's kappa coefficient assesses the consistency between two independent labeling and grouping results. The independent-samples *t*-test or Mann–Whitney U test was used to compare groups of baseline quantitative variables, whereas the χ^2^ test was used to compare qualitative variables. The Wilcoxon signed-rank test was used to compare the RNFL thickness and GCC thickness of total participants at the initial and final visits, and the Mann–Whitney U test was used to compare the five-year changes in RNFL thickness and GCC thickness between the two groups. Bonferroni correction was applied to the corresponding results for RFNL (temporal, superior, nasal, inferior) and GCC (superior, inferior, GCC focal loss volume, GCC global loss volume). Univariate and multivariate regression analyses were performed to identify the associations between changes in inferior RNFL and average GCC thickness with other ocular and systemic parameters. Variables with a *P* < 0.3 in the univariate analysis were included in the multivariate regression model to determine independent predictors. Three outliers were detected in the GCC follow‑up measurements from one subject, all of which were replaced with the mean of adjacent measurements. All *P*-values were two-sided, and the level of statistical significance was set at *P* < 0.05.

## Results

The study included 149 healthy eyes from 149 participants with a 5-year follow-up. The mean age was 59.79 ± 5.61 years (range 50–76 years), and the mean AL was 22.78 ± 0.87 mm (range 20.81–25.28 mm) at baseline. The distribution of AL is shown in Figure 2. The mean age in each group was 60.08 ± 5.56 years in the ONH/PPA changed group and 59.52 ± 5.69 years in the ONH/PPA unchanged group. No statistically significant difference in age distribution was observed between the groups (*P* > 0.05). The baseline ocular characteristics and demographic parameters of all eyes were not significantly different between the two groups (Table 1).

**Figure 1.**
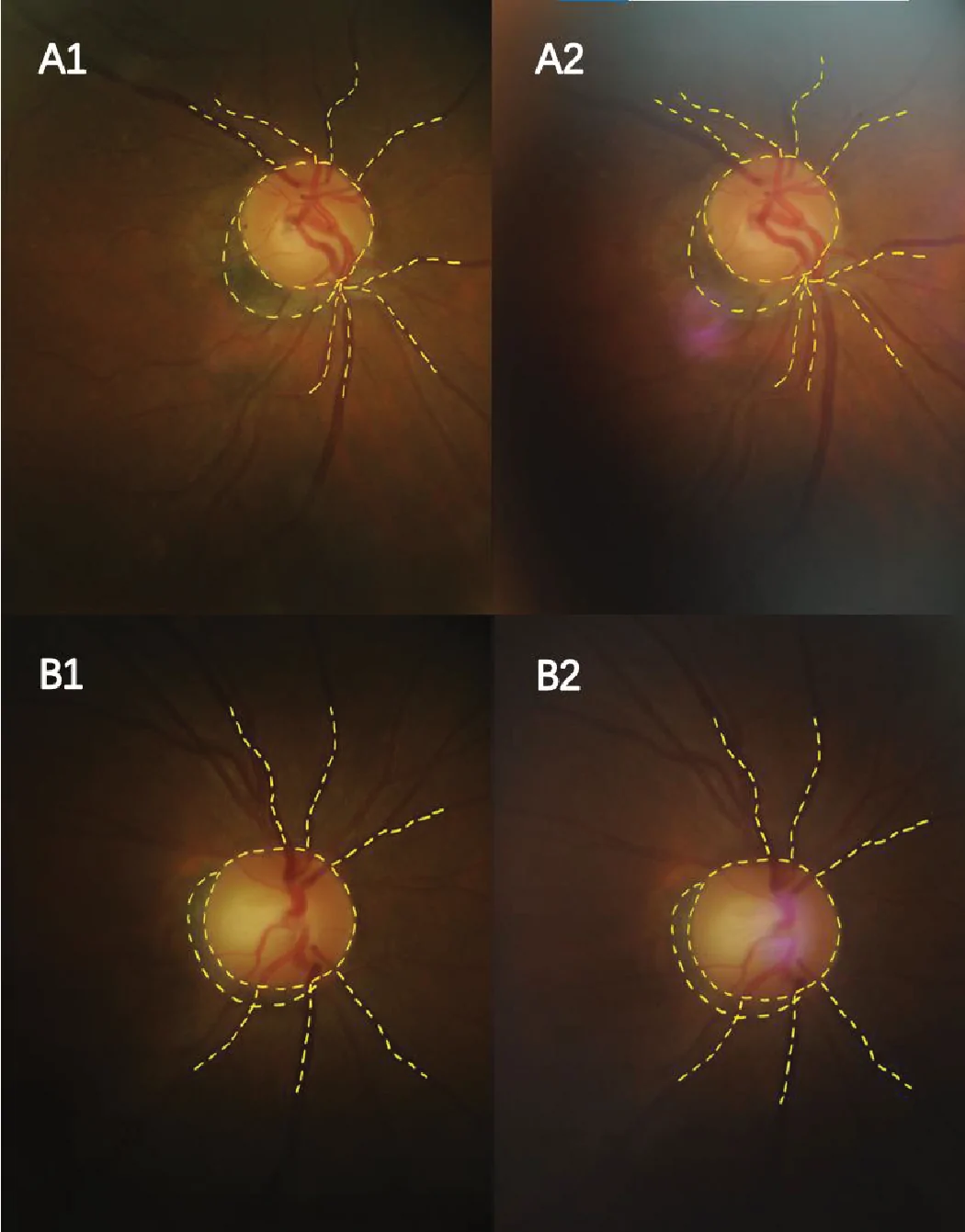
Fundus images showing structural changes in the ONH and peripapillary region. (**A1**, **B1**) Images acquired at the initial visit. (**A2**, **B2**) Images acquired at the final visit. In **A1**, major vessels and the disc margins were extracted from disc photographs (*dashed line*). In **A2**, the extracted boundaries of major vessels and disc margins were superimposed to create a composite follow-up disc image (*dashed line*). **A1** and **A2** represent changes in ONH/PPA, whereas **B1** and **B2** represent no changes in ONH/PPA.

**Figure 2.**
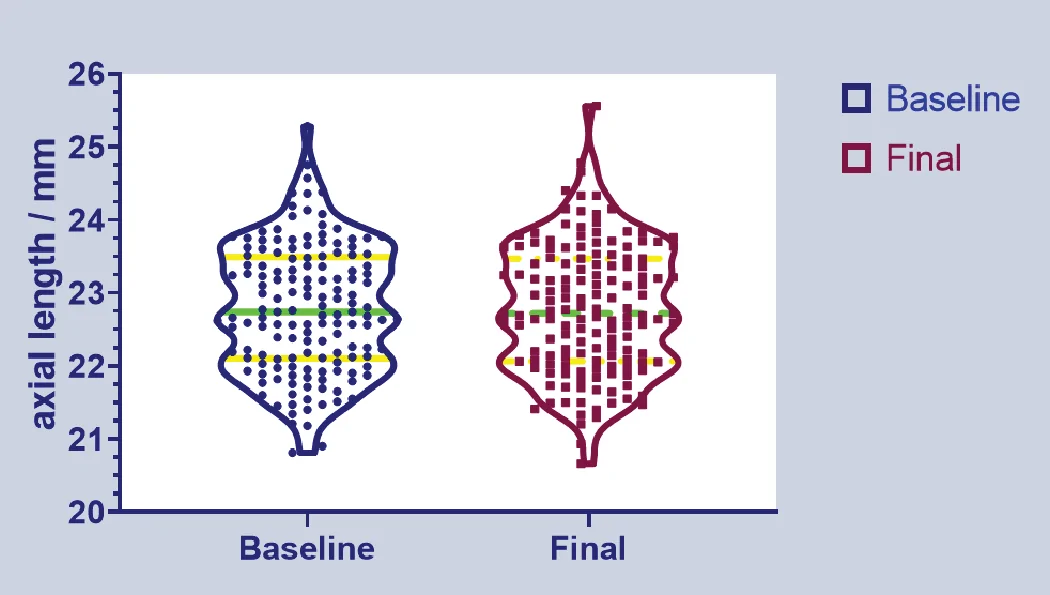
Distribution of axial lengths at baseline and final follow‑up. *Green* and *yellow solid lines* represent the median and interquartile range (IQR) of axial length at baseline, respectively; *green* and *yellow dashed lines* denote the median and IQR of axial length at final follow‑up.

**Table 1.** Demographic and Ocular Characteristics of Subjects in Two Groups

Variables	ONH/PPA Changed Group (*N* = 72)	ONH/PPA Unchanged Group (*N* = 77)	*P* Value
Male	17 (23.6%)	20 (26.0%)	0.74
Baseline age (years)	60.08 ± 5.56	59.52 ± 5.69	0.54
Baseline BMI	28.03 ± 3.99	27.17 ± 3.18	0.15
Baseline SBP (mm Hg)	137.86 ± 17.70	136.05 ± 13.86	0.49
Baseline DBP (mm Hg)	84.89 ± 12.35	82.55 ± 11.62	0.24
Baseline EBP	32 (44.4%)	25 (32.5%)	0.13
Baseline EBG	6 (8.3%)	12 (15.6%)	0.18
Baseline VA (logMAR)	0.10 (0.00, 0.10)	0.00 (0.00, 0.22)	0.60
Baseline IOP (mm Hg)	16.00 (15.00, 18.00)	16.00 (15.00, 18.00)	0.64
Baseline SER (diopter)	0.63 (0.13, 1.25)	0.38 (−0.25, 1.13)	0.15
Baseline CCT (µm)	524.35 ± 28.77	532.05 ± 30.27	0.11
Baseline AL (mm)	21.79 ± 1.83	21.67 ± 1.63	0.67
△AL (mm/5 years)	0.00 (−0.08, 0.06)	0.00 (−0.06, 0.06)	0.47
Baseline VCDR	0.40 (0.05, 0.52)	0.37 (0.20, 0.48)	0.60
Average RNFL thickness (µm)			
Baseline	113.80 ± 14.05	115.24 ± 10.34	0.47
Final	111.77 ± 13.57	114.07 ± 10.95	0.26
Average GCC thickness (µm)			
Baseline	96.38 ± 7.57	96.85 ± 6.89	0.69
Final	95.04 (90.77, 100.23)	95.44 (92.59, 99.52)	0.73

EBG, elevated blood glucose; EBP, elevated blood pressure; IQR, interquartile range; SD, standard deviation.

Statistics presented: mean ± SD; median (IQR).

^*^EBP is defined as SBP >140 mm Hg and/or DBP >90 mm Hg; EBG is defined as random plasma glucose >11.1 mM.

At both baseline and final visits, the inferior quadrant RNFL was thicker than the superior quadrant, followed by the nasal and temporal quadrants (Table 2). The RNFL and GCC thickness showed significant thinning after follow up time of five years. The change in average RNFL thickness was −1.59 ± 3.93 µm, −2.03 ± 4.17 µm, and −1.17 ± 3.66 µm in total participants, the ONH/PPA changed group, and the ONH/PPA unchanged group, respectively (Table 2). For the change in average GCC thickness, it was −1.11 ± 1.99 µm, −1.24 ± 1.99 µm, and −0.98 ± 1.99 µm in total participants, the ONH/PPA changed group, and the ONH/PPA unchanged group, respectively (Table 2). In the two subgroups, significant differences were observed in the change of the inferior RNFL thickness (*P*_adj_ = 0.044), whereas no statistically significant differences were found in the changes of other RNFL and GCC parameters (all *P* > 0.05) (Table 2).

**Table 2.** Comparison of RNFL and GCC Thickness Between Baseline and Final Visits, and Comparison of Changes Between the Two Groups

	Total (*N* = 149)				Change (µm)			
Variables	Mean ± SD/Median (IQR)	*P*1	*P*1 Adjusted	Change (µm)	ONH/PPA Changed Group (*N* = 72)	ONH/PPA Unchanged Group (*N* = 77)	*P*2	*P*2 Adjusted
RNFL thickness (µm)								
Average		**<0.001**	—	−1.59 ± 3.93	−2.03 ± 4.17	−1.17 ± 3.66	0.11	—
Baseline	115.71 (107.59, 123.17)							
Final	113.90 (105.80, 121.29)							
Temporal		**0.002**	**0.008**	−0.92 ± 4.30	−1.15 ± 5.34	−0.71 ± 3.05	0.90	1
Baseline	74.56 (66.40, 79.98)							
Final	73.46 (65.16, 79.21)							
Superior		**0.002**	**0.008**	−1.27 ± 5.26	−1.50 ± 5.69	−1.07 ± 4.85	0.96	1
Baseline	136.84 (122.10, 147.82)							
Final	136.64 (122.90, 146.13)							
Nasal		**<0.001**	**<0.001**	−1.74 ± 6.02	−2.36 ± 5.29	−1.16 ± 6.62	0.09	0.35
Baseline	103.95 (94.43, 113.44)							
Final	102.55 (92.05, 111.29)							
Inferior		**<0.001**	**<0.001**	−2.40 ± 6.32	−3.60 ± 6.30	−1.29 ± 6.17	**0.011**	**0.044**
Baseline	150.37 (140.32, 159.88)							
Final	147.79 (138.13, 158.63)							
GCC thickness (µm)								
Average		**<0.001**	—	−1.11 ± 1.99	−1.24 ± 1.99	−0.98 ± 1.99	0.42	—
Baseline	96.62 ± 7.20							
Final	95.52 ± 7.14							
Superior		**<0.001**	**<0.001**	−1.12 ± 2.23	−1.27 ± 2.29	−0.98 ± 2.19	0.43	1
Baseline	96.13 ± 7.49							
Final	95.01 ± 7.42							
Inferior		**<0.001**	**<0.001**	−1.09± 2.07	−1.21 ± 2.10	−0.97 ± 2.05	0.31	1
Baseline	97.26 (92.99, 101.57)							
Final	95.98 (91.75, 100.69)							
GCC-FLV		**<0.001**	**<0.001**	0.16 ± 0.59	0.21 ± 0.76	0.11 ± 0.35	0.54	1
Baseline	0.81 (0.30, 1.55)							
Final	0.96 (0.38, 1.74)							
GCC-GLV		**<0.001**	**<0.001**	0.45 ± 0.96	0.51 ± 0.99	0.38 ± 0.94	0.41	1
Baseline	1.86 (0.78, 4.11)							
Final	2.31 (1.06, 4.40)							

IQR, interquartile range; SD, standard deviation; *P*1, *P* value between baseline and final values; *P*1 Adjusted, *P*1 value after Bonferroni correction; *P*2, *P* value for the five-year change between the two subgroups; *P*2 Adjusted, *P*2 value after Bonferroni correction; GCC-FLV, GCC focal loss volume; GCC-GLV, GCC global loss volume.

Statistics presented as median (IQR) or mean ± SD. Bonferroni correction was applied to the corresponding results for RFNL (temporal, superior, nasal, inferior) and GCC (superior, inferior, GCC-FLV, GCC-GLV). Values with statistical significance are shown in boldface.

Univariate and multivariate linear regression analyses were used to estimate the independent associations between ocular parameters (baseline VA, baseline IOP, baseline SER, baseline CCT, baseline AL, change in AL, baseline VDR, baseline DA, change in DA, baseline CA, baseline RA, baseline VCDR, change in VCDR, baseline MCD, change in ONH/PPA) and systemic parameters (sex, baseline age, baseline body mass index (BMI), baseline SBP, baseline DBP, baseline pulse rate) and the change in inferior RNFL thickness (Table 3). In the univariate linear regression analysis, the change in inferior RNFL thickness was significantly associated with sex (*P* = 0.079), baseline AL (*P* = 0.095), change in AL (*P* = 0.035), change in DA (*P* < 0.001), baseline VCDR (*P* = 0.236), and change in ONH/PPA (*P* = 0.026) (Table 3). In the multivariate analysis, the change in inferior RNFL thickness was used as the dependent variable, and variables with *P* < 0.3 from the univariate analysis were included in the multivariate linear regression model (Table 3). We then excluded variables that were no longer significantly associated with the change in inferior RNFL thickness. In the multivariate analysis, the change in inferior RNFL thickness was significantly associated with baseline AL (*P* = 0.025), change in AL (*P* = 0.003), change in DA (*P* < 0.001), and change in ONH/PPA (*P* = 0.045), with sex, baseline AL, change in AL, change in DA, baseline VCDR, and change in ONH/PPA included as independent variables.

**Table 3.** The Univariate and Multivariate Analysis of Factors Associated With Changes in Inferior RNFL Thickness

	Univariate Analysis				Multivariate Analysis				
Variables	B	β	95% CI of B	*P* value	B	β	95% CI of B	*P* value	VIF
Sex	2.103	0.144	−0.247, 4.453	**0.079**	0.695	0.048	−1.570, 2.960	0.545	1.125
Baseline age (years)	0.086	0.076	−0.097, 0.269	0.354					
Baseline BMI (kg/m^2)^	0.010	0.006	−0.276, 0.295	0.946					
Baseline SBP (mm Hg)	0.005	0.012	−0.060, 0.070	0.883					
Baseline DBP (mm Hg)	0.008	0.014	−0.078, 0.093	0.862					
Baseline pulse rate	−0.028	−0.052	−0.114, 0.059	0.529					
Baseline VA (logMAR)	3.155	0.082	−3.114, 9.424	0.322					
Baseline IOP (mm Hg)	0.059	0.018	−0.463, 0.582	0.823					
Baseline SER (diopter)	−0.392	−0.071	−1.290, 0.505	0.389					
Baseline CCT (µm)	−0.006	−0.027	−0.040, 0.029	0.743					
Baseline AL (mm)	−0.501	−0.137	−1.091, 0.089	**0.095**	−0.655	−0.179	−1.226, −0.084	**0.025**	1.137
△AL (mm, total)	9.181	0.173	0.643, 17.720	**0.035**	12.357	0.232	4.269, 20.444	**0.003**	1.077
Baseline VDR (mm)	2.069	0.058	−3.768, 7.906	0.485					
Baseline DA (mm^2^)	0.416	0.025	−2.311, 3.144	0.763					
△DA (mm^2^)	16.223	0.331	8.693, 23.754	**<0.001**	16.871	0.345	9.582, 24.160	**<0.001**	1.032
Baseline CA (mm^2^)	−0.609	−0.029	−4.024, 2.806	0.725					
Baseline RA (mm^2^)	1.167	0.058	−2.113, 4.446	0.483					
Baseline VCDR	−2.793	−0.098	−7.431, 1.845	**0.236**	−1.907	−0.067	−6.207, 2.392	0.382	1.053
△VCDR	5.535	0.059	−9.822, 20.892	0.477					
Baseline MCD (mm)	−0.007	−0.085	−0.020, 0.006	0.304					
△ONH/PPA	2.303	0.183	0.285, 4.322	**0.026**	1.894	0.150	0.039, 3.749	**0.045**	1.010

CI, confidence interval; VIF, variance inflation factor.

Values with statistical significance are shown in boldface.

Univariate and multivariate linear regression analyses were used to estimate the independent associations between ocular parameters (baseline VA, baseline IOP, baseline SER, baseline CCT, baseline AL, change in AL, baseline VDR, baseline DA, change in DA, baseline CA, baseline RA, baseline VCDR, change in VCDR, baseline MCD, change in ONH/PPA) and systemic parameters (sex, baseline age, baseline BMI, baseline SBP, baseline DBP, baseline pulse rate) and the change in average GCC thickness (Table 4). In the univariate linear regression analysis, the change in average GCC thickness was significantly associated with baseline SBP (*P* = 0.151), baseline DBP (*P* = 0.136), baseline CA (*P* = 0.037), baseline RA (*P* = 0.214), and baseline MCD (*P* = 0.098) (Table 4). In the multivariate analysis, the change in average GCC thickness was used as the dependent variable, and variables with a *P* < 0.3 from the univariate analysis were included in the multivariate linear regression model (Table 4). We then excluded variables that were no longer significantly associated with the change in average GCC thickness. In the multivariate analysis, the change in average GCC thickness was not significantly associated with any of the parameters (all *P* > 0.05), with baseline SBP, baseline DBP, baseline CA, baseline RA, and baseline MCD included as independent variables.

**Table 4.** The Univariate and Multivariate Analysis of Factors Associated With Changes in Average GCC Thickness

	Univariate Analysis				Multivariate Analysis				
Variables	B	β	95% CI of B	*P* Value	B	β	95% CI of B	*P* Value	VIF
Sex	0.059	0.013	−0.690, 0.807	0.877					
Baseline age (years)	−0.009	−0.027	−0.067, 0.048	0.748					
Baseline BMI	−0.045	−0.081	−0.134, 0.045	0.325					
Baseline SBP (mm Hg)	−0.015	−0.118	−0.035, 0.005	**0.151**	−0.007	−0.054	−0.038, 0.025	0.673	2.404
Baseline DBP (mm Hg)	−0.020	−0.123	−0.047, 0.006	**0.136**	−0.011	−0.066	−0.054, 0.032	0.617	2.588
Baseline pulse rate	0.003	0.021	−0.024, 0.031	0.803					
Baseline VA (logMAR)	0.726	0.060	−1.252, 2.704	0.726					
Baseline IOP (mm Hg)	0.061	0.060	−0.104, 0.225	0.466					
Baseline SER (diopter)	−0.146	−0.084	−0.0.428, 0.137	0.309					
Baseline CCT (µm)	0.000	0.007	−0.010, 0.011	0.934					
Baseline AL (mm)	0.047	0.041	−0.140, 0.235	0.620					
△AL (mm, total)	−1.016	−0.061	−3.742, 1.710	0.462					
Baseline VDR (mm)	0.055	0.005	−1.787, 1.897	0.953					
Baseline DA (mm^2^)	−0.274	−0.052	−1.132, 0.584	0.529					
△DA (mm^2^)	0.116	0.008	−2.398, 2.630	0.927					
Baseline CA (mm^2^)	−1.131	−0.171	−2.192, −0.071	**0.037**	−1.502	−0.227	−3.503, 0.499	0.140	3.523
Baseline RA (mm^2^)	0.650	0.102	−0.380, 1.679	**0.214**	0.464	0.073	−0.684, 1.612	0.426	1.254
Baseline VCDR	−0.613	−0.068	−2.077, 0.852	0.410					
△VCDR	−0.497	−0.017	−5.343, 4.348	0.839					
Baseline MCD (mm)	−0.004	−0.136	−0.008, 0.001	**0.098**	0.003	0.106	−0.006, 0.011	0.518	4.033
△ONH/PPA	0.267	0.067	−0.379, 0.912	0.415					

CI, confidence interval; VIF, variance inflation factor.

Values with statistical significance are shown in boldface.

## Discussion

RNFL and GCC parameters have been widely used in the early diagnosis of glaucoma^15^; however, ONH and PPA may change during follow-up in healthy eyes, such as optic nerve tilt or PPA enlargement. It remains uncertain whether these structural changes affect longitudinal RNFL and GCC measurements. This study provides a long-term longitudinal evaluation of RNFL and GCC thickness progression in healthy eyes using spectral-domain OCT. The results of our study indicate that both RNFL and GCC thickness decreased over the five-year follow-up period in healthy eyes. This reduction was observed not only in the group with ONH/PPA changes, but also in the group without such changes. These findings are consistent with previous research.^16^ Moreover, we found that inferior RNFL thinning was associated with changes in ONH/PPA, whereas average GCC thinning was not. Bilgin et al.^17^ quantified RNFL in adult offspring of patients with primary open-angle glaucoma (POAG) and reported significantly reduced RNFL thickness—most pronounced in the inferior quadrant. Potential contributors to the pronounced RNFL thinning observed in the inferior quadrant include: first, limited sample size, which may affect statistical power and generalizability—thus warranting validation in larger, prospective cohorts; second, as shown by the results of this study, the RNFL gradually thins with age in healthy eyes, and the initial thinning may occur in the inferior quadrant.

A five-year longitudinal study investigating RNFL changes in patients with multiple sclerosis (with or without optic neuritis) included 40 healthy eyes in the control group and reported an average RNFL thickness reduction of 2.2 µm over time.^18^ Another prospective study on multiple sclerosis included 29 participants in the normal control group and found that the average change in RNFL thickness over a two-year follow-up period was 2.2 µm.^19^ In our study, average RNFL thickness decreased by 1.59 µm over a five-year follow-up period, a reduction smaller than that reported in the two aforementioned studies. This discrepancy may be attributed to differences in sample size, measurement instruments, and the racial composition of the study populations. With regard to average GCC thickness, our study observed a reduction of 1.11 µm over the 5-year period. Kang et al.^20^ conducted a longitudinal assessment of GCC thickness in individuals with physiological large disc cupping, including 269 eyes with normal intraocular pressure and normal visual fields, and reported a 1.3 µm reduction in GCC thickness over a five-year follow-up. Similarly, our findings indicate that the reduction in GCC over a 5-year follow-up period was smaller than that reported by Kang et al.,^20^ likely attributable to differences in sample size and the characteristics of the included study populations.

Leung et al.^21^ examined the impact of age-related changes in macular and circumpapillary RNFL measurements on the assessment of glaucoma progression. They observed that such changes can be detected in normal eyes and have implications for the analysis of glaucoma progression. Therefore identifying reliable indicators for early glaucoma detection during follow-up of healthy eyes is of paramount importance. The diagnostic ability of RNFL and GCC in glaucoma has been analyzed in several studies using the area under curve (AUC).^22^^,^^23^ With aging, physiological remodeling occurs in the ONH and its peripapillary structures. In this study, we compared the longitudinal associations of ONH and peripapillary structural parameters with RNFL and GCC thickness measurements over a five-year follow-up period to identify metrics that are relatively robust to age-related anatomical variation. We found a significant difference in the change of inferior RNFL between the two subgroups, whereas no statistically significant differences were observed in the change for other RNFL and GCC parameters. Furthermore, univariate and multivariate linear regression were conducted to evaluate the influencing factors of inferior RNFL and average GCC thickness. The results indicate that inferior RNFL parameters are more sensitive than GCC parameters to changes in ONH structure during follow-up examinations in healthy eyes. Dhabarde et al.^23^ demonstrated that both GCC thickness and RNFL thickness exhibit comparable diagnostic efficacy in detecting early glaucoma. Barua et al.^22^ reported that inferior GCC yielded the highest AUC (0.827) for distinguishing POAG from healthy individuals. Although this difference did not reach statistical significance relative to other parameters (*P* > 0.05), the authors noted the potential utility of inferior GCC in detecting early glaucoma. Our findings revealed that changes in inferior RNFL were influenced by baseline AL, changes in AL, changes in DA, and ONH/PPA structural variations (Table 3), whereas average GCC parameters showed no significant association with these variables. It is evident that AL and ONH structure-related parameters have a substantial influence on changes in inferior RNFL thickness. Furthermore, a longitudinal observational study of 196 eyes from 123 patients with POAG found that GCC may be more useful than RNFL for assessing disease progress in advanced glaucoma.^24^ Since Zeimer et al.^25^ proposed that changes in retinal thickness within the macula may serve as an indicator of glaucoma status, macular parameters have increasingly been suggested as a viable alternative or complementary approach to peripapillary RNFL thickness for glaucoma diagnosis. In this study cohort, GCC parameters appear to be unrelated to morphological changes of the ONH and PPA, which may facilitate the early detection of glaucoma and its progression in individuals with age-related ONH/PPA alterations.

This study has several limitations. First, all participants were Chinese individuals aged ≥50 years, and both ethnic and age-related factors may influence RNFL and GCC changes in healthy eyes. Second, participants with hypertension or diabetes were not excluded, and these conditions are recognized as potential risk factors for reductions in RNFL and GCC thickness. Demir et al.^26^ investigated RNFL and GCC thickness in patients with type 2 diabetes mellitus and reported reduced RNFL and GCC thickness relative to healthy controls; however, these reductions did not reach statistical significance. Similarly, no statistically significant difference was observed between the two subgroups in the baseline proportions of elevated blood pressure and elevated blood glucose (Table 1). Third, the study cohort comprised 37 male participants (24.8%) and 112 female participants (75.2%). A prior study reported marginally higher mean RNFL thickness in female participants, whereas male participants exhibited modestly greater mean ganglion cell layer (GCL) and GCL plus inner plexiform layer thicknesses.^27^ They reported no statistically significant association between RNFL thickness in either eye and sex; similarly, GCL thickness in the right eye showed no significant association with sex. In contrast, superior and average GCL thicknesses in the left eye were significantly associated with sex. Therefore further investigation is warranted to clarify the relationship between RNFL and GCC thicknesses and sex. Fourth, this study conducted only a qualitative analysis of ONH/PPA morphological changes and did not perform quantitative assessments on the cases with alterations; this will be addressed and improved in future research.

## Conclusions

Both RNFL and GCC thinning were detectable in healthy eyes over five years. This study demonstrated that inferior RNFL thinning was associated with AL and ONH structural parameters, whereas no significant association was observed between average GCC thinning and these variables. Our study results indicate that GCC measurements are more resistant to interference in detecting changes in ONH morphology and PPA anatomy compared to RNFL measurements, and GCC may aid in the early detection of glaucoma and its progression in individuals with age-related changes in the ONH and PPA region.
